# A predictor for predicting *Escherichia coli* transcriptome and the effects of gene perturbations

**DOI:** 10.1186/1471-2105-15-140

**Published:** 2014-05-13

**Authors:** Maurice HT Ling, Chueh Loo Poh

**Affiliations:** 1 School of Chemical and Biomedical Engineering, Nanyang Technological University, Nanyang Ave, Singapore, Singapore

## Abstract

**Background:**

A means to predict the effects of gene over-expression, knockouts, and environmental stimuli *in silico* is useful for system biologists to develop and test hypotheses. Several studies had predicted the expression of all *Escherichia coli* genes from sequences and reported a correlation of 0.301 between predicted and actual expression. However, these do not allow biologists to study the effects of gene perturbations on the native transcriptome.

**Results:**

We developed a predictor to predict transcriptome-scale gene expression from a small number (n = 59) of known gene expressions using gene co-expression network, which can be used to predict the effects of over-expressions and knockdowns on *E. coli* transcriptome. In terms of transcriptome prediction, our results show that the correlation between predicted and actual expression value is 0.467, which is similar to the microarray intra-array variation (p-value = 0.348), suggesting that intra-array variation accounts for a substantial portion of the transcriptome prediction error. In terms of predicting the effects of gene perturbation(s), our results suggest that the expression of 83% of the genes affected by perturbation can be predicted within 40% of error and the correlation between predicted and actual expression values among the affected genes to be 0.698. With the ability to predict the effects of gene perturbations, we demonstrated that our predictor has the potential to estimate the effects of varying gene expression level on the native transcriptome.

**Conclusion:**

We present a potential means to predict an entire transcriptome and a tool to estimate the effects of gene perturbations for *E. coli*, which will aid biologists in hypothesis development. This study forms the baseline for future work in using gene co-expression network for gene expression prediction.

## Background

One of the key challenges in systems biology is to develop a complete computational model of biology that can be used for both integration of knowledge and to develop and test hypotheses. A number of computational tools had been developed (reviewed in [1]) over the years, such as COBRA toolkit [2]. However, Medema et al. [1] did not mention about any tools for transcriptome prediction. Selinger et al. [3] proposed that a means to predict gene expressions will be useful for predicting the effects of gene over-expression, knockouts, and environmental stimuli.

A number of recent studies had attempted to predict gene expression using *in silico* methods. Chikina et al. [4] used microarray data to predict tissue-specific gene expression in various tissues of *Caenorhabditis elegans*. Ouyang et al. [5] used transcription factors binding data from ChIP-seq experiments to predict gene expression in mouse embryonic cells. McLeay et al. [6] expanded on Ouyang et al. [5] by modeling the binding efficiency of transcription factors to promoters; thereby, using it to predict gene expressions. McLeay et al. [6] reported correlation of 0.64 when tested on GM12878 cells but histones modification and chromatin accessibility data needs to be incorporated, which may limit its application due to the lack of required data. Fox and Erill [7] used relative codon usage bias to predict the expression levels of *E. coli* genes of more than 1000 bp, achieving a correlation of 0.489 between predicted and actual expression. This is higher than the correlation of 0.301 reported by Roymondal et al. [8] when correlating relative codon usage bias to the expression levels of *E. coli* genes of all lengths. A further study by the same group attempted to predict the expression of *Synechocystis* PCC 6803 (a cyanobacterium) using relative codon usage bias reported a correlation between 0.240 and 0.356 [9]. However, there had been no study demonstrating the use of gene co-expression network (GCN) in gene expression prediction in *E. coli*.

GCN had been commonly used to study expressional similarities of genes [10], where the nodes are the genes and a link (an edge) between 2 nodes when the gene-pair is co-expressed. The basis of GCN is that expressionally correlated genes are likely to be functionally related [11,12] or evolutionarily conserved [13,14]. GCN had been successfully used in several cases, such as identifying developmental processes [15], annotating functional genes [16], and studying disease progression [17]. Although there had been a number of methods proposed to estimate the degree of co-expression [18]; such as using rank correlation [19], weights [20] and mixed-models [21]; Pearson’s correlation is commonly used [4,11,22,23] due to presence of upper and lower boundaries of correlation coefficient, resulting in ease of interpretation [24]. Once the co-expression between two genes is established, the expression level of a gene can be predicted from the known expression of another gene by means of linear regression [25]. This suggests that GCN has the advantage of estimating a large number of gene expressions from a small number of known gene expressions.

In this study, we developed a predictor to predict transcriptome-scale gene expression from a small number of known gene expressions using GCN, which may be used to predict the effects of over-expressions and knockdowns on *E. coli* transcriptome. The correlation of 21 genes that are detected by 2 probes on the microarray is 0.490. Using microarray data not used in GCN building, our transcriptome prediction results show that the correlation between expected and predicted expressions using expression values is 0.467. Our perturbation prediction results show that the correlation between predicted and actual expression values among perturbation-affected genes to be 0.698. Using our ability to predict the effects of gene perturbations, we presented a case study to estimate the effects of varying gene expression level of *hydrogenase 2 maturation endopeptidase* (*hybD*); thereby, identifying a range of expression levels in which there is no effect on the native transcriptome and we termed this range as expressional buffer. Hence, this study presents a potential means to estimate transcriptome-scale gene expressions which has the potential to predict the effects of gene over-expression, knockouts, and environmental stimuli [3].

## Results and discussion

We developed a predictor based on GCN to predict transcriptome-scale gene expression and estimate the effects of changing the expression of genes, such as over-expression and under-expression, on a native transcriptome.

### Fifty-nine source genes reach 6140 genes

A total of 51,121,216 permuted probe-pairs were generated from 10,112 non-control probes in GPL3154. These non-control probes were mapped to 10,091 genes. Thus, only 21 genes were represented by 2 probes (given in Additional file 1: Table S2). For simplicity, we shall use "genes" to represent both "genes" and "probes" hereafter. The average correlation of these gene-pairs is 0.027, which is similar to that reported in other studies [14,26]. Using the correlation threshold suggested by Reverter et al. [27] of absolute correlation coefficient that is higher than 0.75 (p-value = 1.28e-102 after Bonferroni correction), only 533,311 (1.04%) pairs and 7,360 (72.78%) genes remained and were used to construct the co-expression network.

Using the 21 genes that were represented by 2 probes on the microarray, intra-array variation [28] can be estimated by analyzing the differences from these 2 probes [29]. Theoretically, their expression values will be the same and the ratio of expression values will be 1, which can be translated to perfect correlation, as they are measuring the same transcript. Using all 605 microarrays, our results suggest that the average correlation is 0.490 with a standard error of 0.0488.This is similar to the correlation of 0.535 (p-value = 0.36, power > 0.99) reported by Ling et al. [14] on microarray technical replicates of identical biological samples. The average deviation [ $\left(\sum_{i=1}^{N}|\text{average}\;\text{ratio}-1|\right)/N$] from a perfect ratio of 1 is 19.19%, suggesting that the average intra-array variation can be estimated to be 19.19% (Additional file 1: Table S2). Our estimate falls within 11% and 33% intra-array variation estimated by Anderson et al. [30] whom proposed a novel Array Microenvironment Normalization (AMN) to reduce 72% of the intra-array variation. However, Gyorffy et al. [31] demonstrated that results from RMA (Robust Multi-array Average) normalization correlates well with both tissue samples and cell lines even though other normalization schemes appears to work better with tissue samples or cell lines independently. In addition, AMN has not been shown to correlate well with quantitative PCR results. Hence, considering that RMA normalization correlates well with both tissue samples and cell lines, we chose to continue with RMA normalized data. Nevertheless, our estimated intra-array variation of 19.19% suggests potential area of future studies in normalization techniques aiming at reducing such variation as intra-array variation represents noise in the source data which may affect downstream analyses [32].

**Figure 1:**
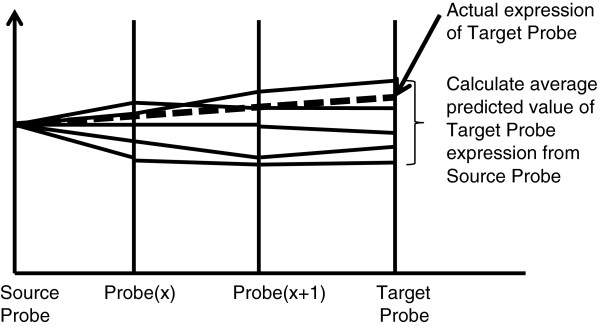
**Concept in predicting target gene expression value.** Target gene expression value can be estimated from a single source gene expression based on linear regression. In this figure, the source probe/gene and target probe/gene are separated by 2 sets of probe/genes and 5 different paths. Expression values of probes/genes adjacent to the source probe/gene [denoted as Probe(x)] can be estimated by linear regression. This can be done repeatedly to reach the target. Target probe/gene expression value can then be statistically inferred.

**Figure 2:**
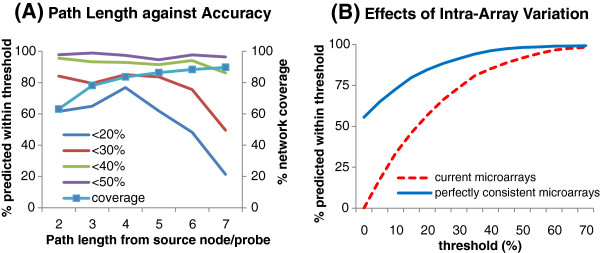
**Precision of prediction.** Panel **A** shows the percentage of genes predicted within specific thresholds from 2 to 7 jumps from the source gene. The predicted value of each gene is defined as the average predicted values from source gene to target gene across different paths. If the actual expression value of a gene is +/- 20% the predicted value, then the gene is considered to be predicted within 20% threshold. The network coverage from 392 source genes (see Figure 3) is shown. Panel **B** shows the difference in prediction precision between the current set of microarray with 19.19% intra-slide variation and a set of theoretically consistent microarray with no intra-slide variation.

After GCN construction, the next step was to determine a small set of genes with the maximum network coverage and minimum degree of separation (also known as jump) as network coverage is directly proportional to the extent of predictable transcriptome and the error in prediction is directly proportional to the number of jumps. We analyzed the number of jumps between any given gene-pairs. Our results suggest that the density peaks at 4 jumps (Additional file 1: Figure S1). With reference to Figure 1, when a pair of genes is linked by a finite number of paths, the expression of one of the pairs (known as a target gene) can be predicted if expression of the other gene (known as a source gene) is known. As there can be many paths between the source and target genes, there can be many predicted expression values for the target gene as the number of predicted values equals the number of paths. Our results show that accuracy at 20% error decreases drastically from path length of 5 or more (Figure 2A), suggesting that the limits of predictability is 4 jumps. Although it can be argued that path lengths of 2 or 3 may yield higher accuracy, the number of source genes will increase as the number of source genes needed is inversely proportional to the path length in order to achieve the same network coverage. In addition, our results also suggest that intra-array variation adversely affect prediction accuracy (Figure 2B).

**Figure 3:**
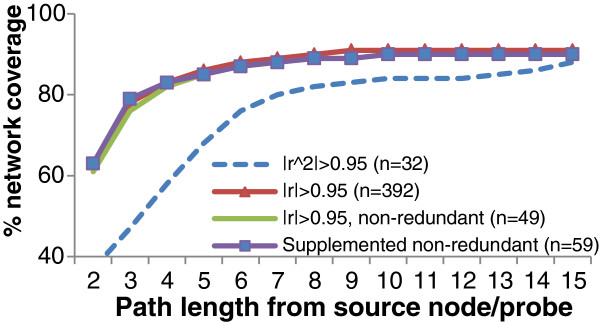
**Network coverage by path length.** The number of target genes reachable by 32 source genes (from a threshold of |r^2^| > 0.95) is much lesser than that reachable by 392 source genes (from a threshold of |r| > 0.95) at a path length of 4. However, using 392 source genes is not practical for experimental validation. By removing redundant source genes, we arrived at 49 source genes but at a loss of 101 target genes. By supplementing with 10 genes with high degrees (total of 59 source genes), we increased the reach by 87 target genes, giving a total of 6140 reachable target genes from 59 source genes.

We analyzed the degree of network coverage using 2 sets of source probes – a set of 32 source genes from coefficient of determination (r^2^) of more than 0.95 and a set of 392 source genes from absolute Pearson’s correlation of more than 0.95. Since coefficient of variation can be used as a measure of prediction accuracy between a pair of source and target probes, strong correlation in the first jump is likely to improve the overall prediction. Our results show that the coverage from the set of 392 genes is significantly better than that of 32 genes (Figure 3). However, 392 is a large number of genes to measure experimentally. We analyzed this set of 392 genes in order to reduce it into a smaller set of marker genes [33] which is feasible for experimental work. At 4 jumps, a number of these genes reach to the same set of target genes. By removing redundancy, we reduced 392 genes to 49 genes but the coverage dropped from 6154 to 6053 genes. We examined the set of genes not reached by these 49 source genes within 4 jumps and added 10 genes with the highest degree (most number of edges) to increase the number of source genes from 49 to 59. With this addition, the coverage increases to 6140 genes. We argue that adding more source probes at this stage is unlikely to give equivalent increase in coverage. Hence, we proceeded with a set of 59 source genes (see Additional file 1: Table S3 for the description of these 59 genes).

### Transcriptome predicted within 40% error using 59 source genes

We attempted to predict *E. coli* transcriptome using the panel of 59 source genes. To do so, we implemented a single pass transcriptome predictor where each target gene will be predicted using expression value from one source gene. We evaluated the accuracy of our predictor using a set of 30 microarrays from experiments not used in the GCN construction (see Additional file 1: Table S4 for the microarrays used). These sets of microarray data had been published in 25 different experimental studies [34-58]; thus, representing a set of unbiased data for evaluating the performance of our predictor (see Additional file 1: Table S4 for details of experiments). In this aspect, we hold the same evaluation principles as Abadia et al. [59], whom used data from various centres worldwide to evaluate the performance of a newly developed protocol.

Although these 30 microarrays originated from a diverse range of studies, several recent studies [60-62] had suggested that published microarray datasets contain value beyond their initial studies. For example, several studies had analyzed published microarray datasets for reference genes [63,64] and other biologically significant features [65]. Moreover, most of the 30 microarrays originated from studies that were representative of the type of experimental studies which we expect our predictor to be useful in. For example, Traxler et al. [56] examined the global effects of amino acid starvation in *E. coli* MG1655 and Lee et al. [48] examined the expression of *E. coli* stress-related proteins in the presence of pollutants. Hence, our evaluation also represented 30 experimental case studies on the use of our transcriptome predictor.

Our results suggest a positive correlation between the average predicted expression values and the actual expression values of each target gene across all 30 transcriptomes (average correlation = 0.467, standard error (SE) = 0.0383, p-value = 2.77e-13). This is similar to the correlation of 0.489 (p-value = 0.656) reported by Fox and Erill [7] using relative codon usage bias to predict the expression levels of *E. coli* genes of more than 1000 bp and higher than the reported correlation of 0.240 to 0.356 (p-value < 0.031) in a study using codon usage bias to predict expression of *Synechocystis* PCC 6803 genes [9]. As our predictor is not restricted to the length of gene that can be predicted as in the case of Fox and Erill [7], the correlation of 0.301 between actual and predicted expression reported by Roymondal et al. [8] is a more accurate comparison to our result as Roymondal et al. [8] use relative codon usage bias to predict the expression levels of all *E. coli* genes instead of those more than 1000 bp. Based on this, our predictor is more accurate (p-value = 0.0002) than that reported by Roymondal et al. [8]. In addition, this is not significantly different from the correlation of 0.490 between duplicate probes from the microarray data measuring the same transcript (p-value = 0.613), suggesting that intra-array variation accounts for a substantial portion of transcriptome prediction error.

**Figure 4:**
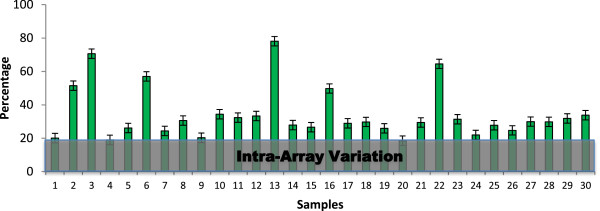
**Prediction evaluation test for single-pass transcriptome predictor.** Thirty microarrays were used to evaluate the accuracy of the single-pass transcriptome predictor (see Additional file 1: Table S3 for the microarrays used). The number of paths between the source and target genes is 30 to 10000. Error bar denotes standard error.

**Figure 5:**
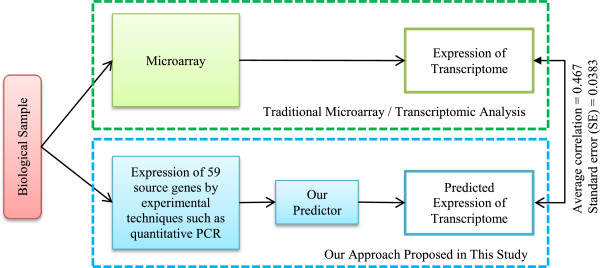
**Comparison between our approach (this study) and traditional microarray experiments for analyzing transcriptome.** Our approach (this study) demonstrates the ability to predict the expression of the entire transcriptome from the expression of 59 genes; thus, presenting our approach as an economical initial screening of hypotheses before more costly experimental techniques, such as microarrays.

Our results show that 24 of the 30 transcriptomes (see Additional file 1: Table S4 for the microarrays used) are predicted within 40% error using 30 to 10000 paths between each source and target gene (Figure 4), with the average error of 34.29% (standard error = 2.807%). This is comparable to 33% error (p-value = 0.65) using chromatin states and transcription factor occupancy, which are less readily available than gene expression values, to predict spatial-temporal expression of genes [66]. Hence, our predictor can potentially be used as a preliminary *in silico* hypothesis screening tool, which only requires the expression of a panel of source genes and can be obtained with routine experimental tools such as quantitative PCR, prior to full-scale transcriptome analysis (Figure 5).

Many studies use experimental techniques, such as PCR-based techniques, on a small set of genes to validate microarray results. Kendall et al. [45] use quantitative PCR for detailed analysis of microarray findings elucidated by comparing the transcriptomes of wild-type *E. coli* 86–24 strain and *luxS* mutant VS94, which corresponds to GSM180104 and GSM180102 respectively. These 2 microarrays have not been used in our GCN construction. Hence, we predicted the transcriptomes of *E. coli* 86–24 strain and *luxS* mutant VS94 using source gene expressions from GSM180104 and GSM180102 respectively. We compare our prediction results with 10 quantitative PCR results of Kendall et al. [45], showing 3 of the 10 evaluated genes to be differentially expressed. Our results suggest 8 out of 10 matched conclusions with one false positive and false negative each (Additional file 1: Table S5).

Although our results show that the predicted gene expressions of 30 representative test samples are more accurate than that of Roymondal et al. [8], our results also show that only 24 of the 30 transcriptomes can be predicted within 40% error and 8 out of 10 findings using our prediction match quantitative PCR results of Kendall et al. [45]. Despite using 30 representative test samples for our evaluation, our results are based on meta-analysis of published data. Using meta-analysis of published experimental data, we have shown the potential of the predictor. However, the protocol will need to be further validated using more condition-specific experiments. At the moment, our study forms a baseline towards this direction.

It is conceivable that using more than one source gene to predict a target gene may improve prediction accuracy. To test this hypothesis, we developed a multi-pass transcriptome predictor that allows for the use of any number of source genes to predict a target gene. Network coverage analysis shows that 59 source genes can reach a total of 169,012 genes in 4 jumps or each target gene is reached by an average of 27.5 source probes. This suggests that the computation time for multi-pass transcriptome prediction will be 27.5 times longer than single pass transcriptome prediction if maximum number of source gene per target gene is used.

**Figure 6:**
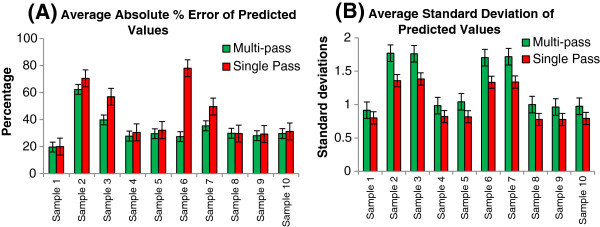
**Prediction evaluation test for multi-pass transcriptome predictor.** Ten microarrays which had been used for single-pass transcriptome predictor evaluation were used to evaluate multi-pass transcriptome predictor (see Additional file 1: Table S3 for the microarrays used). Error bar denotes standard error. Panel **A** shows the average percent difference between the predicted and actual target gene values. Panel **B** shows the average standard deviation from each predicted values across different paths.

Ten of the 30 transcriptomes used in the evaluation of single pass transcriptome predictor were used to evaluate the multi-pass transcriptome predictor (see Additional file 1: Table S4 for the microarrays used). Our results suggest that there is no difference in terms of percentage difference (Figure 6A, p-value = 0.076) even though 3 of the 10 predicted transcriptomes (Samples 3, 6, and 7) are significantly less accurate when predicted by multi-pass method. By examining the standard deviations of the predicted values of each target gene (Figure 6B), multi-pass method consistently gives higher standard deviation compared to single pass method (p-value = 3.10e-5). This suggests that better prediction by multi-pass method in terms of average standard deviations between expected and predicted expression levels of target genes is an artifact as a result of larger standard deviations for the predicted values of each target gene. Correlation between expected and predicted target expression values is significant (average correlation = 0.269, SE = 0.0455, p-value = 1.13e-4), which is similar to that previously reported [8,9] but lower than that reported by Fox and Erill [7] and significantly lower from our single pass prediction (p-value = 0.003, power = 0.999). This suggests that multi-pass transcriptome predictor does not give better prediction compared to single pass transcriptome predictor despite requiring significantly more computational time.

### 83% of perturbation-affected genes predicted within 40% error

An important application of transcriptome prediction model is predicting the effects of gene over-expression, knockouts, and environmental stimuli *in silico*[3]. Over-expressions and knockdowns or under-expressions are collectively known as perturbations. A recent study [67] had modeled the protein concentrations leading to G2 cell cycle checkpoint and validated their simulations of protein level perturbations with published studies.

Our predictor has the potential to estimate the effects of gene perturbation(s). For example, if geneA is over-expressed by 2 times, the affected genes will be the set of genes reachable within 4 jumps of geneA. Our predictor uses a microarray sample as a background transcriptome and performs two predictions. The first prediction predicts the expression values of all reachable genes from the genes of interest before perturbation. Perturbations are carried out by varying the expression values of the genes of interest before predicting the expression values all reachable genes from the genes of interest after perturbation. Both predictions will provide a predicted value (the mean) and a standard deviation of the affected probes, which allow for standard hypothesis testing and power analysis to be performed.

For evaluation, we identified a background transcriptome, a test transcriptome, and perturbed one or more genes from the background transcriptome to the value of the test transcriptome. Experimentally, if the effects of a 2 times over-expression of geneA in *E. coli* were to be studied, the standard experimental protocol will require an over-expression of geneA using a vector which regulates the expression of geneA under an inducible promoter and compare the transcriptomes of the control sample against the over-expressed sample [68,69]. In our study, the background and test transcriptomes were selected to represent the control and perturbed samples respectively. Three replicates were performed on each of the 6 evaluation tests including single, double and quadruple gene perturbations (see Additional file 1: Table S6 for setup details).

**Figure 7:**
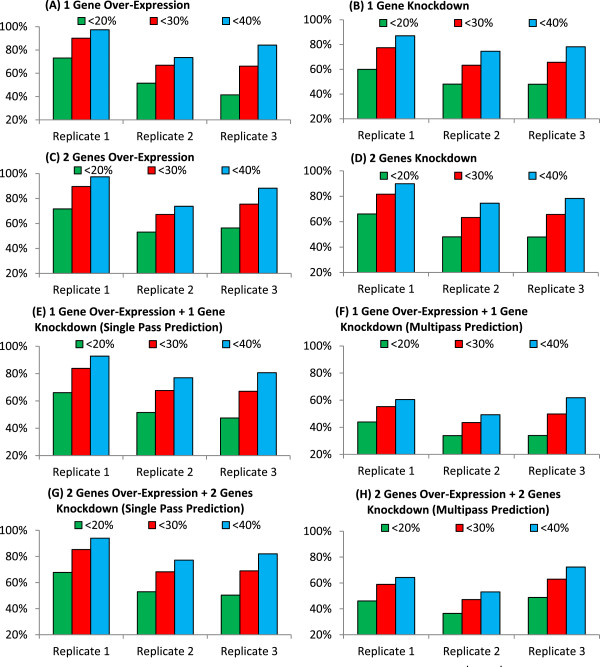
**Prediction evaluation test for perturbation transcriptome predictor.** The vertical axis shows the percentage of affected genes predicted within a specific threshold. Actual percentages are displayed within each bar. Panels **A**, **B**, **C** and **D** are single gene over-expression, single gene knockdown or under-expression, double gene over-expression and double gene knockdown respectively. Panels **E** and **F** are two gene perturbations, single gene over-expression and single gene knockdown, predicted using single pass and multi-pass respectively. Panels **G** and **H** are four gene perturbations, double gene over-expression and double gene knockdown, predicted using single pass and multi-pass respectively.

Our results show that at least 73.6% of the affected genes in single gene over-expression or knockdown are predicted within 40% error (Figure 7A and B). For double gene over-expression or knockdown, at least 73.8% of the affected genes are predicted within 40% of error (Figure 7C and D). Using single pass prediction, our results show that at least 77.0% of the genes affected by single gene over-expression with single gene knockdown (2 genes perturbed; Figure 7E) and at least 77.2% of the genes affected by double gene over-expression with double gene knockdown (4 genes perturbed; Figure 7G) can be predicted within 40% error. Hence, our results suggest that an average of 83.4% (SE = 0.195%) of perturbation-affected genes can be potentially predicted within 40% error (which can also be interpreted as within 1.4 folds accuracy).

Comparing single pass versus multi-pass prediction (Figure 7E versus 7F, and 7G versus 7H), accuracy between the predicted and actual expression values of the affected genes dropped when multi-pass prediction was used. Statistical comparison between single and multi-pass method shows that this difference is significant (p-value = 0.0012). This is consistent with the findings in our initial multi-pass predictor evaluation. The average correlation between the expression values of affected genes predicted by single-pass method after perturbation is 0.698 with a standard deviation of 0.123 (Additional file 1: Table S7), which is significant (p-value = 7.44e-15). This result is comparable to the correlation of 0.64 (p-value = 0.062) reported by McLeay et al. [6], using ChIP-seq, histones and DNase scores to predict gene expression in mammalian cells. This suggests expression values of genes affected by perturbations can be potentially predicted with accuracy comparative to next generation sequencing methods and sequence analyses. This suggests that our predictor may be a useful *in silico* tool to examine gene perturbations.

Hence, our evaluation also presents itself as a case study of how this predictor can be used. For example, the second replicate of single gene knockdown evaluation corresponds to 56% knockdown of *hydrogenase 2 maturation endopeptidase* (*hybD*), involving in the maturation of hydrogenase 2. Of the 1603 genes affected by this perturbation, 77 genes are directly correlated and 27 genes show more than 3x differences between background expression level and predicted expression level after perturbation. Of the 1526 genes affected between 2 to 4 jumps, 60 are significantly different after Bonferroni correction between predicted expression level before and after perturbation. These 87 genes were analyzed for Gene Ontology enrichment using GOEAST [70]. All 5 significant molecular functions enriched were of carbon/sugar transferase-typed activity (GOIDs 0008194, 0008378, 0035250, 0016757, and 0016758). This agrees with recent findings associating hydrogenase 2 to hydrogen production during glucose [71] or glycerol fermentation [72].

### Expression buffer of *hydrogenase 2 maturation endopeptidase (hybD)*

Knowing that 56% knockdown of *hybD* has an impact on the native transcriptome, it is plausible to consider the question of expression buffer. That is, how much expressional variation of *hybD* must occur before the underlying native transcriptome is affected? In this case study, we explore this question on a background of *E. coli* MG1655 pure culture (GSM663167).

**Figure 8:**
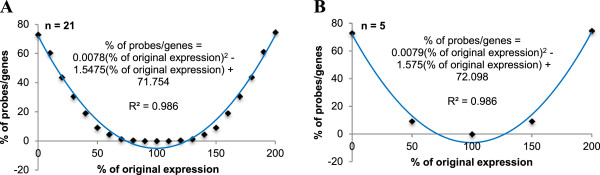
**Percentage of reachable genes affected by varying levels of hydrogenase 2 maturation endopeptidase (hybD) expressions.** Panel **A** shows 10% stepwise expression variation from total knockout to 2x over-expression (21 data points). Panel **B** shows 50% stepwise expression variation from total knockout to 2x over-expression (5 data points). Both models are not statistically different from each other (paired t-test p-value = 0.385).

Using 10% stepwise perturbation of *hybD* from knockout to 2x over-expression (Figure 8A), our results suggest that the number of affected genes is symmetrical and fits a quadratic model (paired t-test p-value = 0.182, r^2^ = 0.986). By solving the roots of the quadratic model, we estimate that an expression between 73.88% and 124.52% of the original expression (microarray intensity = 8.535) does not affect the transcriptome of *E. coli* MG1655. In addition, our results also suggest that a reduced model of 5 data points (Figure 8B) is a good estimate (paired t-test p-value = 0.385), suggesting the possibility to reduce computational time if a large number of perturbation analyses are needed. The reduced model estimated an expression buffer between 71.21% and 128.15% of 8.535.

Hence, the predictor may be used to provide estimation to a research question proposed by Selinger et al. [3] – what are the effects of 50% versus 60% knockdown of *hybD*? Our results suggest that 148 genes are affected when *hybD* is knocked down by 50% (50% of original expression) compared to 307 genes when *hybD* is knocked down by 60% (40% of original expression).

### This study provides baseline and test cases for future work

In this study, we present a potential means to predict virtually the entire transcriptome from a set of 59 source genes, which may be useful for synthetic biologists to predict the effects of transgene [33]. In addition, our predictor has the potential to examine the effect of one or more genes when their expression is/are changed [3] and shown to perform comparatively to previous studies on predicting prokaryotic gene expressions using sequence features such as codon usage bias [7,9].

Using the simplest statistical model to relate the expression values of 2 genes, this study acts as a baseline for future work. Non-linear or higher-order regression models [18-21], may be used to improve prediction accuracy. The prediction accuracy may also be improved with additional microarray data as they come online or including data from less noisy sources, such as from RNA sequencing. At the same time, we had described the test cases used (see Additional file 1) throughout this study, which can be used to evaluate future improvements to this work.

## Conclusion

In this study, we demonstrate that the transcriptome of *E. coli* can be potentially predicted from a set of marker gene expressions or from known perturbation. The former enables thousands of gene expressions to be predicted from a small set of known gene expressions while the latter enables *in silico* evaluation of the effects gene perturbation(s) such as gene over-expression(s) and/or under-expression(s). Hence, we present a potential means to predict an entire transcriptome and a tool to estimate the effects of gene perturbations for *E. coli*, which will aid biologists in hypothesis development. This study forms the baseline for future work in using gene co-expression network for gene expression prediction.

## Methods

### Construction of co-expression network and regression models

The CEL files of 605 *E. coli* microarrays across 40 experiments were downloaded from NCBI Gene Expression Omnibus (see Additional file 1: Table S1 for a list of series used) and RMA normalized using Affymetrix Expression Console. Pairwise permutations of Pearson’s correlation were calculated and the expression values for the pair of genes were fitted into first order linear regression equation in the form of Gene(x) = b_1_Gene(y) + b_0_. Pairs with absolute Pearson’s correlation of more than 0.75 were retained for building co-expression network using *NetworkX* where the nodes were the genes and an edge existed between the nodes when the absolute Pearson’s correlation between the two genes was more than 0.75.

### Predicting transcriptome

Two transcriptome predictors, single pass and multi-pass, were implemented. The difference between the two predictors is that the single pass predictor performed one prediction per target gene whereas the multi-pass predictor allowed a target gene to be predicted using 2 or more source genes. Thus, in single pass prediction, a target gene expression will be estimated from one or more paths from the source gene expression. Once a target gene expression is estimated, its expression will not be re-estimated even though the target gene can be predicted by more than one source gene. The sequence of target gene expression prediction is dependent on the sequence of source gene expression and the number of jumps (degree) from the source gene. For example, if a target gene can be estimated by 2 different source genes at 3 degrees, the first source gene will be used to estimate the target gene expression in single pass predictor. If a target gene can be estimated by 2 different source genes at 3 and 4 degrees respectively, the target gene expression will be estimated by the source gene at 3 degrees instead of the source gene at 4 degrees in single pass predictor. In multi-pass predictor, both source genes will be used to estimate the target gene expression regardless of positional sequence of the source gene list or the degrees between source genes and the target gene. Given a list of source genes (marker genes) and their expression values, the transcriptome predictors predict all genes reachable within 4 jumps using a loop over the linear regression models. For example, if Gene(A) is a source gene with known expression and is connected to Gene(C) via Gene(B), then the expression of Gene(B) can be predicted by the known expression of Gene(A) by linear regression between Gene(A) and Gene(B). Bringing this a step forward, the expression of Gene(C) can be predicted by the predicted expression of Gene(B) by linear regression between Gene(B) and Gene(C). Therefore, the expression level of Gene(C) can be predicted as Gene(C) = b_1,B-C_(b_1,A-B_Gene(A) + b_0,A-B_) + b_0,B-C_ where b_1,A-B_ and b_0,A-B_ is the first-order linear regression gradient and intercept between Gene(A) and Gene(B) respectively, and b_1,B-C_ and b_0,B-C_ is the first-order linear regression gradient and intercept between Gene(B) and Gene(C) respectively. As there could be more than one path between any source and target genes via different intermediary genes, there could be more than one predicted expression values. The predictor would report the arithmetic mean and standard deviation of the predicted values (see Figure 1).

### Predicting the effects of perturbation(s)

A list of perturbations was given as ratio of the original expression values, for example, 1.8 times of Gene(A) and 0.4 times of Gene(B). The predictor estimated the effects of perturbations by a two-pass transcriptome prediction where the first pass predicted the expression values of all affected target genes within 4 jumps using the original expression values of the background transcriptome [1× Gene(A) and 1× Gene(B)], followed by a second pass using the perturbed values from the background transcriptome [1.8× Gene(A) and 0.4× Gene(B)]. As a result, each perturbation runs using different combinations of perturbed genes might have different numbers of affected target genes.

### Evaluating predictors

The single pass and multi-pass transcriptome predictors were evaluated using 30 and 10 microarrays that were not used for model building respectively (see Additional file 1: Table S3 for arrays used and labeling). Perturbation prediction was evaluated using six types of perturbations (1. Single gene over-expression. 2. Single gene knockdown. 3. Double gene over-expression. 4. Double gene knockdown. 5. Single gene over-expression with single gene knockdown. 6. Double gene over-expression with double gene knockdown.) on 3 replicates (see Additional file 1: Table S4 for detailed setup and microarrays used). For each microarray, the expression values of 59 genes were extracted and used as source genes to predict all reachable genes, known as target genes, within 4 jumps. The target genes consisted of adjacent genes (one jump from source genes) and non-adjacent genes (two to four jumps from source genes). As there would be only one path from source gene to adjacent gene, standard deviation would not be calculated and only non-adjacent genes would be used to evaluate the predictors. The accuracy of prediction was determined by the number of standard deviations and the percentage difference between the expected expression value (from the microarray data) and the average predicted values.

## Competing interests

The authors declare that they have no competing interests.

## Authors’ contributions

ML and CLP conceived of the project. ML performed the research and analyzed the data. CLP provided essential comments and guidance. The manuscript was written by ML and edited by CLP. All authors read and approved the final manuscript.

## Supplementary Material

Additional file 1: Table S1 Microarray data series for model building. **Table S2.** Internal consistency of microarray values. **Table S3.** List of 59 source probes. **Table S4.** Microarrays used to evaluate the accuracy of single pass and multi-pass transcriptome predictors. **Table S5.** Comparison between quantitative PCR findings of Kendall et al. [45] and gene expression prediction. **Table S6.** Perturbation predictor evaluation setup. **Table S7.** Correlations between predicted and expected expression values of genes affected by perturbation(s). **Figure S1.** Distribution of path length. Click here for file
