# Supplementary material for: A predictor for predicting Escherichia coli transcriptome and the effects of gene perturbations
Source: BMC Bioinformatics. 2014 May 13;15:140. doi: 10.1186/1471-2105-15-140 (PMC4038595; doi:10.1186/1471-2105-15-140)
Supplement: Additional file 1: Table S1 — Microarray data series for model building. Table S2. Internal consistency of microarray values. Table S3. List of 59 source probes. Table S4. Microarrays used to evaluate the accuracy of single pass and multi-pass transcriptome predictors. Table S5. Comparison between quantitative PCR findings of Kendall et al. [45] and gene expression prediction. Table S6. Perturbation predictor evaluation setup. Table S7. Correlations between predicted and expected expression values of genes affected by perturbation(s). Figure S1. Distribution of path length. [file 1471-2105-15-140-S1.docx]

# Additional file 1

**Table S1. Microarray data series for model building.** 605 microarrays from the following 40 data series in NCBI Gene Expression Omnibus (GEO) were used for model building. The respective CEL files were downloaded and processed by Affymetrix Expression Console using RMA normalization.

| **Microarray Series ID** | **Title / Citation (if available)** |
| --- | --- |
| GSE35371 | Genome-wide transcription analysis of Escherichia coli in response to extremely low-frequency magnetic fields.  Citation: Bioelectromagnetics 2012, 33(6):488-96. |
| GSE30639 | Evolution of gene expression during long term coexistence in a bacterial evolution experiment.  Citation: Proc Natl Acad Sci U S A 2012, 109(24):9487-92. |
| GSE34023 | Overexpression of BglJ and LeuO in Escherichia coli K12.  Citation: Mol Microbiol 2012, 83(6):1109-23. |
| GSE17526 | Transcriptional responses of Escherichia coli rpoS- BW25113 vs. wild-type BW25113 under 15% ethanol shock in log phase. |
| GSE15406 | Expression data from E. coli cells overexpressing GraL for short and long periods of time.  Citation: Nucleic Acids Res 2010, 38(5):1636-51. |
| GSE17519 | Transcription analysis of E. coli O157:H7 when exposed to sodium benzoate |
| GSE10345 | Genome-wide analysis of transcriptional termination in E. coli.  Citation: Science 2008, 320(5878):935-8. |
| GSE3665 | Global transcriptional machinery engineering in E. coli in the presence and absence of ethanol.  Citation: Metab Eng 2007, 9(3):258-67. |
| GSE31140 | E. coli response to antimicrobial arylamides.  Citation: Antimicrob Agents Chemother 2011, 55(11):5043-53. |
| GSE17465 | Transcriptional profile of Escherichia coli K12 strain JM109 harboring pMG1 and pMG1-IrrE under 1M NaCl shock |
| GSE14238 | Expression data from E. coli O157:H7 growth in the absence or presence of HEp-2 epithelial cells.  Citation: PLoS One 2009, 4(3):e4889. |
| GSE7656 | E. coli GeneChip study of E. coli responses to osmotic and heat stresses.  Citation: J Bacteriol 2008, 190(10):3712-20. |
| GSE10159 | Expression of Escherichia coli treated with cefsulodin and mecillinam, alone at the minimum inhibitory concentration.  Citation: J Bacteriol 2008, 190(6):2065-74. |
| GSE6992 | Expression data from a paraquat time course experiment in wild type and SoxR deficient strains.  Citation: PLoS One 2007, 2(11):e1186. |
| GSE39607 | Expression data from QdNOs treated Escherichia coli |
| GSE12554 | Transcript Analysis of E. coli ATCC 35218 suggest a Role of cranberry Juice in Inhibiting the growth of E. coli.  Citation: Biochem Biophys Res Commun 2008, 377(3):992-4. |
| GSE17584 | Transcriptional effects of CRP* expression in Escherichia coli.  Citation: J Biol Eng 2009, 3:13. |
| GSE21652 | Expression data for transcriptional engineering mutants capable of L-tyrosine overproduction.  Citation: Proc Natl Acad Sci U S A 2012, 109(34):13538-43. |
| GSE34275 | Escherichia coli K-12 grown in the presence of glycerol exhibits upregulation in genes for acid stress chaperones and concomitant downregulation in genes involved in generation of acidic metabolites |
| GSE28795 | Expression data from E. coli cells overexpressing either GreA or GreB in ppGpp0 cells in the dksA+ or dksA- background.  Citation: J Bacteriol 2012, 194(2):261-73. |
| GSE29440 | Design of an improved host platform for the over expression of recombinant proteins in Escherichia coli.  Citation: AMB Express 2011, 1(1):33. |
| GSE28412 | Analyzing the metabolic stress response of recombinant Escherichia coli cultures expressing human interferon beta in high cell density fed batch cultures using time course transcriptomic data.  Citation: AMB Express 2011, 1(1):33. |
| GSE21839 | Transcriptome analysis of wild type E. coli (K-12 MG1655) comparing to mutant E. coli strain (ECOM4) under aerobic and anaerobic conditions.  Citation: Appl Environ Microbiol 2010, 76(19):6529-40. |
| GSE15500 | Analysis of differences in gene expression due to small adaptive mutations in RNA polymerase B' subunit (rpoC).  Citation: Proc Natl Acad Sci U S A, 107(47):20500-5. |
| GSE17420 | Global effect of RpoS on gene expression in pathogenic Escherichia coli O157:H7 strain EDL933.  Citation: BMC Genomics 2009, 10:349. |
| GSE15533 | Expression data from E. coli O157.  Citation: Appl Environ Microbiol 2009, 75(19):6110-23. |
| GSE13589 | Gene expression of E. coli MG1655 pOX38Km at the outside and inside of biofilms.  Citation: Biotechnol Bioeng 2009, 103(5):975-83. |
| GSE29486 | Uncoupling growth and product formation kinetics to design improved strains for recombinant protein production in Escherichia coli |
| GSE15050 | Bacterial adrenergic signaling.  Citation: PLoS Pathog 2009, 5(8):e1000553. |
| GSE28399 | Transcriptional responses of uropathogenic E. coli to stresses caused by sodium chloride or urea.  Citation: Infect Immun 2013, 81(1):80-9. |
| GSE20095 | Antagonistic regulation of motility and other cellular functions by RpoN and RpoS in Escherichia coli.  Citation: Mol Microbiol 2011, 79(2):375-86. |
| GSE20374 | Metabolic and transcriptional response to cofactor perturbations in Escherichia coli.  Citation: J Biol Chem 2010, 285(23):17498-506. |
| GSE14796 | Comparison of gene expression profiles of E. coli W3110 normal colonies vs. L-form colonies.  Citation: PLoS One 2009, 4(10):e7316. |
| GSE4724 | Transcriptome analysis of the arginine regulon in E. coli. Citation: Microbiology 2006, 152(Pt 11):3343-54. |
| GSE40693 | Transcriptional response of Escherichia coli O157:H7 to cinnamaldehyde. Citation: Appl Environ Microbiol 2013, 79(3):942-50. |
| GSE40648 | Effect of simulated microgravity on E. coli K12 MG1655 growth and gene expression.  Citation: PLoS One 2013, 8(3):e57860. |
| GSE24524 | Identification of genes induced on nitrate, role of OxyR.  Citation: Science 2012, 336(6080):470-3. |
| GSE33895 | Enterohemorrhagic Escherichia coli (EHEC)8624 double deletions of qseC and qseE grown in DMEM.  Citation: Infect Immun 2012, 80(2):688-703. |
| GSE12831 | The role of qseE, qseF and qseG in the regulation of EHEC virulence.  Citations: Proc Natl Acad Sci U S A 2009, 106(14):5889-94; Microbiology 2010, 156(Pt 4):1167-75. |
| GSE13562 | Effects of sidA and AHL on EHEC virulence.  Citation: Proc Natl Acad Sci U S A 2010, 107(21):9831-6. |

**Table S2. Internal consistency of microarray values.** In GPL3154 platform, 21 ORFs have 2 probes. As these 2 probes are detecting the same transcript, the ratio of these 2 probes across all 605 microarrays used in model building (see Table S1) can be used to evaluate the consistency and reproducibility of the microarray data. The ratio of 1 represents perfect consistency between the values of the 2 probes. **Main findings: On the whole, the average ratio of each pairs of probe deviates from perfect consistency by 19.19% with a standard deviation of 12.61%, suggesting that the intra-variation in each microarray is estimated to be 19.19%.**

| **Locus Tag** | **Probe IDs in GPL3154** | **Average Ratio** | **Standard Deviation of Ratio** |
| --- | --- | --- | --- |
| c0009 | 1764627_at; 1762903_s_at | 1.3038 | 0.2115 |
| c0047 | 1768525_x_at; 1764568_s_at | 1.3963 | 0.2368 |
| c0270 | 1762715_s_at; 1759141_x_at | 0.9472 | 0.1899 |
| c0471 | 1765415_s_at; 1760620_x_at | 1.1954 | 0.1998 |
| c0500 | 1765162_x_at; 1760746_s_at | 0.7720 | 0.1735 |
| c1008 | 1768502_x_at; 1759784_s_at | 1.0249 | 0.1055 |
| c1036 | 1769172_s_at; 1761935_x_at | 0.7546 | 0.1663 |
| c1062 | 1764489_s_at; 1759858_x_at | 1.0428 | 0.0738 |
| c1279 | 1767962_s_at; 1761636_x_at | 0.9270 | 0.1173 |
| c1406 | 1768745_x_at; 1759358_s_at | 1.2334 | 0.2411 |
| c1423 | 1764990_x_at; 1764540_s_at | 0.9114 | 0.2324 |
| c1538 | 1767181_s_at; 1764970_x_at | 1.0941 | 0.2074 |
| c2119 | 1768125_s_at; 1763077_x_at | 1.3623 | 0.4420 |
| c2375 | 1762395_x_at; 1759795_s_at | 0.8361 | 0.1339 |
| c3100 | 1765970_x_at; 1760913_s_at | 1.3756 | 0.3529 |
| c3668 | 1766683_x_at; 1760009_s_at | 0.8965 | 0.1644 |
| c3878 | 1762755_x_at; 1760349_s_at | 1.0820 | 0.2689 |
| c4250 | 1765614_x_at; 1764554_s_at | 0.9105 | 0.1311 |
| c4575 | 1767118_x_at; 1766121_s_at | 1.2759 | 0.1452 |
| c5447 | 1767177_s_at; 1766758_at | 0.5783 | 0.1136 |
| Z2146 | 1766728_x_at; 1761853_s_at | 0.8227 | 0.2864 |

**Table S3. List of 59 source probes.** Probe ID and Locus Tag are Probe IDs in the microarray (GPL3154) and locus tag in NCBI gene record respectively.

| **Probe ID** | **Locus Tag** |  | **Probe ID** | **Locus Tag** |  | **Probe ID** | **Locus Tag** |
| --- | --- | --- | --- | --- | --- | --- | --- |
| 1763376_at | b4022 |  | 1761072_at | b2757 |  | 1764010_s_at | Z5054 |
| 1768602_at | b3021 |  | 1763355_at | b2755 |  | 1763879_s_at | Z5056 |
| 1765970_x_at | c3100 |  | 1760568_s_at | b3077 |  | 1759435_at | b3715 |
| 1768855_at | b2182 |  | 1765285_s_at | b0504 |  | 1767485_at | b0780 |
| 1762662_s_at | Z0599 |  | 1762875_at | b2038 |  | 1766425_s_at | b4201 |
| 1764355_at | b2627 |  | 1762326_at | b1141 |  | 1759255_at | b3217 |
| 1761298_at | b2622 |  | 1768785_s_at | ECs3324 |  | 1763325_at | b0056 |
| 1767293_at | b2631 |  | 1759869_at | c5401 |  | 1764978_at | b2638 |
| 1766730_at | b0280 |  | 1766040_at | b3022 |  | 1766452_at | b0057 |
| 1761324_at | b2350 |  | 1760045_s_at | b2487 |  | 1764869_s_at | Z1353 |
| 1762892_s_at | Z1498 |  | 1761673_at | c1544 |  | 1764915_s_at | Z6060 |
| 1763332_s_at | b3393 |  | 1763754_at | c5164 |  | 1768426_s_at | Z1323 |
| 1764533_s_at | b2851 |  | 1767770_at | ECs1067 |  | 1762728_s_at | Z5087 |
| 1766506_s_at | c4687 |  | 1768126_at | c1545 |  | 1767138_s_at | b3678 |
| 1765366_s_at | b2453 |  | 1763914_s_at | Z6074 |  | 1767987_x_at | ECs1229 |
| 1767220_at | c4452 |  | 1764068_x_at | Z3306 |  | 1766777_s_at | ECs1091 |
| 1765015_at | b3623 |  | 1768400_s_at | Z5868 |  | 1763925_s_at | Z3189 |
| 1766029_at | b3629 |  | 1763222_x_at | ECs1124 |  | 1762374_s_at | Z5452 |
| 1760408_s_at | Z5055 |  | 1768805_s_at | Z1153 |  | 1767386_at | b2352 |
| 1767335_s_at | Z1145 |  | 1767009_s_at | Z1144 |  |  |  |

**Table S4. Microarrays used to evaluate the accuracy of single pass and multi-pass transcriptome predictors.** 30 and 10 arrays were used to test single pass and multi-pass transcriptome predictors respectively. The sample numbers correspond to the sample numbers in Figure 4 and 5. For comparison between single pass and multi-pass transcriptome predictors, the sample numbers from single pass transcriptome predictor test were relabeled in Figure 5 (and multi-pass transcriptome predictor test). For example, GSM239043 was used as Sample 3 in and single pass transcriptome predictor test, but the same data from and single pass transcriptome predictor test was relabeled as Sample 2 for and multi-pass transcriptome predictor test in Figure 5.

| **GSM ID** | **Reference**  **(Published Study)** | **Sample Number in Single Pass Transcriptome Prediction Test** | **Sample Number in Multi-Pass Transcriptome Prediction Test** |
| --- | --- | --- | --- |
| GSM174666 | Li et al. (2007) | Sample 1 | Sample 1 |
| GSM180112 | Kendall et al. (2007) | Sample 2 |  |
| GSM239043 | Bansal et al. (2008) | Sample 3 | Sample 2 |
| GSM247667 | Lee et al. (2008) | Sample 4 |  |
| GSM272064 | Hensley et al. (2012) | Sample 5 |  |
| GSM322122 | Reading et al. (2010) | Sample 6 | Sample 3 |
| GSM344445 | Lee et al. (2010) | Sample 7 |  |
| GSM351297 | Nobre et al. (2009) | Sample 8 | Sample 4 |
| GSM355063 | Kim et al. (2010) | Sample 9 |  |
| GSM460022 | Nakanishi et al. (2011) | Sample 10 |  |
| GSM469137 | Moon and Gottesman (2009) | Sample 11 | Sample 5 |
| GSM490263 | Durand and Storz (2010) | Sample 12 |  |
| GSM511647 | Habdas et al. (2010) | Sample 13 | Sample 6 |
| GSM538623 | Strader et al. (2011) | Sample 14 |  |
| GSM543293 | Yang et al. (2012) | Sample 15 |  |
| GSM554443 | Bansal et al. (2012) | Sample 16 | Sample 7 |
| GSM585505 | Traxler et al. (2011) | Sample 17 |  |
| GSM585515 | Traxler et al. (2011) | Sample 18 | Sample 8 |
| GSM622787 | Waters et al. (2011) | Sample 19 |  |
| GSM654474 | Cho et al. (2011) | Sample 20 |  |
| GSM663159 | Chu et al. (2012) | Sample 21 | Sample 9 |
| GSM720471 | unpublished | Sample 22 |  |
| GSM754995 | Chen et al. (2012) | Sample 23 | Sample 10 |
| GSM760804 | Chattopadhyay et al. (2013) | Sample 24 |  |
| GSM768839 | Alteri et al. (2011) | Sample 25 |  |
| GSM352906 | Ma and Wood (2009) | Sample 26 |  |
| GSM511652 | Habdas et al. (2010) | Sample 27 |  |
| GSM585510 | Traxler et al. (2011) | Sample 28 |  |
| GSM632642 | Hidalgo et al. (2011) | Sample 29 |  |
| GSM755000 | Chen et al. (2012) | Sample 30 |  |

**Table S5. Comparison between quantitative PCR findings of Kendall et al. (2007) and gene expression prediction.** Kendall et al. (2007) validated their microarray results using quantitative PCR between 2 *E. coli* strains, 86-24 and VS94. These correspond to GSM180104 and GSM180102 respectively. We extracted the source gene expressions to predict the respective transcriptomes and used 95% confidence after Bonferroni correction as a threshold for significance. Our results show that 8 of the 10 findings using our predictor matched that of Kendall et al. (2007). “NS” denotes “not significant”.

| **Probe ID** | **Locus ID** | **Findings from Kendall et al. (2007)** | **Findings Based on Prediction from This Study** | **Classification** |
| --- | --- | --- | --- | --- |
| 1767609_s_at | Z5132 | NS | NS | True negative |
| 1767809_s_at | Z5126 | NS | NS | True negative |
| 1761005_s_at | Z5120 | NS | NS | True negative |
| 1769270_s_at | Z5107 | P < 0.0005 | P = 1.3E-18 | True positive |
| 1759781_s_at | Z1464 | NS | P = 1.9E-07 | False positive |
| 1761446_s_at | Z5213 | NS | NS | True negative |
| 1761810_s_at | Z5214 | NS | NS | True negative |
| 1761530_s_at | Z2379 | NS | NS | True negative |
| 1762469_s_at | Z4971 | P < 0.005 | P = 1.2E-06 | True positive |
| 1767523_s_at | Z5223 | P < 0.005 | NS | False negative |

**Table S6. Perturbation predictor evaluation setup.** Six types of perturbations were performed (1. Single gene over-expression. 2. Single gene knockdown. 3. Double gene over-expression. 4. Double gene knockdown. 5. Single gene over-expression with single gene knockdown. 6. Double gene over-expression with double gene knockdown.) Each perturbation was performed on three microarrays as triplicates.

| **Type** | **Replicate** | **Setup: Background Transcriptome, Tester, and Perturbation(s)** |
| --- | --- | --- |
| Single Gene Over-Expression | 1 | Background transcriptome: GSM174666  Tester transcriptome: GSM174667  Perturbation(s): Probe 1764761_s_t 🡺 1.69x |
|  | 2 | Background transcriptome: GSM460022  Tester transcriptome: GSM460023  Perturbation(s): Probe 1760847_at 🡺 2.87x |
|  | 3 | Background transcriptome: GSM663156  Tester transcriptome: GSM663166  Perturbation(s): Probe 1768256_at 🡺 2.84x |
| Single Gene Knockdown | 1 | Background transcriptome: GSM174666  Tester transcriptome: GSM174667  Perturbation(s): Probe 1764576_at 🡺 0.66x |
|  | 2 | Background transcriptome: GSM460022  Tester transcriptome: GSM460023  Perturbation(s): Probe 1759383_s_at 🡺 0.435x |
|  | 3 | Background transcriptome: GSM663156  Tester transcriptome: GSM663166  Perturbation(s): Probe 1762322_x_at 🡺 0.435x |
| Double Gene Over-Expression | 1 | Background transcriptome: GSM174666  Tester transcriptome: GSM174667  Perturbation(s): Probe 1764761_s_t 🡺 1.69x  Probe 1760195_s_at 🡺 1.62x |
|  | 2 | Background transcriptome: GSM460022  Tester transcriptome: GSM460023  Perturbation(s): Probe 1760847_at 🡺 2.87x  Probe 1766792_at 🡺 2.87x |
|  | 3 | Background transcriptome: GSM663156  Tester transcriptome: GSM663166  Perturbation(s): Probe 1768256_at 🡺 2.84x  Probe 1759907_s_at 🡺 2.80x |
| Double Gene Knockdown | 1 | Background transcriptome: GSM174666  Tester transcriptome: GSM174667  Perturbation(s): Probe 1764576_at 🡺 0.66x  Probe 1765655_s_at 🡺 0.682x |
|  | 2 | Background transcriptome: GSM460022  Tester transcriptome: GSM460023  Perturbation(s): Probe 1759383_s_at 🡺 0.435x  Probe 1764276_s_at 🡺 0.465x |
|  | 3 | Background transcriptome: GSM663156  Tester transcriptome: GSM663166  Perturbation(s): Probe 1762322_x_at 🡺 0.435x  Probe 1768023_s_at 🡺 0.485x |
| Single Gene Over-Expression with  Single Gene Knockdown | 1 | Background transcriptome: GSM174666  Tester transcriptome: GSM174667  Perturbation(s): Probe 1764761_s_t 🡺 1.69x  Probe 1764576_at 🡺 0.66x |
|  | 2 | Background transcriptome: GSM460022  Tester transcriptome: GSM460023  Perturbation(s): Probe 1760847_at 🡺 2.87x  Probe 1759383_s_at 🡺 0.435x |
|  | 3 | Background transcriptome: GSM663156  Tester transcriptome: GSM663166  Perturbation(s): Probe 1768256_at 🡺 2.84x  Probe 1762322_x_at 🡺 0.435x |
| Double Gene Over-Expression with  Double Gene Knockdown | 1 | Background transcriptome: GSM174666  Tester transcriptome: GSM174667  Perturbation(s): Probe 1764761_s_t 🡺 1.69x  Probe 1760195_s_at 🡺 1.62x  Probe 1764576_at 🡺 0.66x  Probe 1765655_s_at 🡺 0.682x |
|  | 2 | Background transcriptome: GSM460022  Tester transcriptome: GSM460023  Perturbation(s): Probe 1760847_at 🡺 2.87x  Probe 1766792_at 🡺 2.87x  Probe 1759383_s_at 🡺 0.435x  Probe 1764276_s_at 🡺 0.465x |
|  | 3 | Background transcriptome: GSM663156  Tester transcriptome: GSM663166  Perturbation(s): Probe 1768256_at 🡺 2.84x  Probe 1759907_s_at 🡺 2.80x  Probe 1762322_x_at 🡺 0.435x  Probe 1768023_s_at 🡺 0.485x |

**Table S7. Correlations between predicted and expected expression values of genes affected by perturbation(s).** Correlations are calculated from all the affected genes by comparing the perturbation affected expression against the respective test transcriptome. Higher correlation is indicative of the higher predictability. The average correlation coefficient of single-pass method is 0.698 (SD = 0.123). The average correlation coefficient of multi-pass method is 0.392 (SD = 0.036). The difference between single and multi-pass method is significant (p-value = 7.45e-12) using t-test for unequal variance. In addition, paired t-test between the correlations of single and multi-pass method for single/double gene over-expression/knockdown (n = 6) is significant (p-value = 0.0012).

| **Type of Perturbation** | **Replicate 1** | **Replicate 2** | **Replicate 3** | **Average Correlation** |
| --- | --- | --- | --- | --- |
| Single gene over-expression | 0.900 | 0.425 | 0.762 | 0.696 |
| Single gene knockdown | 0.827 | 0.598 | 0.686 | 0.703 |
| Double gene over-expression | 0.865 | 0.446 | 0.759 | 0.690 |
| Double gene knockdown | 0.800 | 0.598 | 0.686 | 0.695 |
| Single gene over-expression + Single gene knockdown (single pass) | 0.847 | 0.545 | 0.708 | 0.700 |
| Single gene over-expression + Single gene knockdown (multi-pass) | 0.463 | 0.360 | 0.339 | 0.387 |
| Double gene over-expression + Double gene knockdown (single pass) | 0.845 | 0.555 | 0.709 | 0.703 |
| Double gene over-expression + Double gene knockdown (multi-pass) | 0.391 | 0.389 | 0.410 | 0.397 |

**Figure S1. Distribution of path length.** Path lengths (number of jumps or degrees of separation) between each pair of genes were computed. Some pairs of genes were not reachable by each other (infinite jumps). Only reachable pairs were used for tabulation.
